# Flow-Enhanced Photothermal Spectroscopy

**DOI:** 10.3390/s22197148

**Published:** 2022-09-21

**Authors:** Ulrich Radeschnig, Alexander Bergmann, Benjamin Lang

**Affiliations:** Institute of Electrical Measurement and Sensor Systems, Graz University of Technology, 8010 Graz, Austria

**Keywords:** gas sensing, photothermal spectroscopy, Fabry–Pérot interferometer, PTS sensors

## Abstract

Photothermal spectroscopy (PTS) is a promising sensing technique for the measurement of gases and aerosols. PTS systems using a Fabry–Pérot interferometer (FPI) are considered particularly promising owing to their robustness and potential for miniaturization. However, limited information is available on viable procedures for signal improvement through parameter tuning. In our work, we use an FPI-based PTS configuration, in which the excitation laser irradiates the target collinearly to the flowing gas. We demonstrate that the generated thermal wave, and thus the signal intensity, is significantly affected by the ratio between excitation modulation frequency and gas flow velocity towards another. We provide an analytical model that predicts the signal intensity with particular considerations of these two parameter settings and validate the findings experimentally. The results reveal the existence of an optimal working regime, depending on the modulation frequency and flow velocity.

## 1. Introduction

Photothermal spectroscopy (PTS) is a promising sensing method that enables the detection and measuring of gases and aerosols down to trace gas levels [1,2,3,4]. Its fundamental principle comprises the excitation of a target substance by absorption of radiation (e.g., a laser beam) and the measurement of the refractive index (RI) change produced. The RI change stems from the thermally induced density variations in the exposed medium and exhibits values typically in the order of 10${}^{-9}$ to 10${}^{-12}$ RI units [2,5]. For the quantification of these changes, PTS uses interferometric techniques. The earliest application of this method for measuring low-concentrated gaseous samples was published in the early 1980s using a Mach–Zehnder interferometer [6]. To date, many additional PTS instruments have been published, with which a wide variety of gas types have been sensed down to ppb and ppt levels, e.g., nitrogen dioxide [7,8], carbon dioxide [9] and many others [10,11,12,13].

Among the most promising photothermal interferometry concepts are those based on the hollow-core fiber [14] and the Fabry–Pérot principle [2], both capable of complementing excellent sensitivity with robustness. Hollow-core fiber systems are limited for real-world application by modal interference, a strong attenuation of light in the fiber and considerably long gas exchange times due to narrow fiber diameters [2,15]. In contrast, PTS systems, which combine a Fabry–Pérot interferometer (FPI) with a compact gas cell, offer a broadly applicable, miniaturizable instrument that exhibits a fast response due to a substantially reduced gas exchange time [2,7,8].

Compared to similar techniques such as resonant photoacoustic spectroscopy, PTS allows for greater freedom in adapting several parameters without the need to change the cell geometry. Important parameters are the excitation modulation frequency and the gas flow rate, which enable tailoring towards a preferred performance characteristic such as sensitivity, signal-to-noise ratio, limit of detection and response time.

In this work, we investigate the influence of the gas flow rate on the signal intensity of an FPI-based PTS sensor. Our system features a downsized photothermal (PT) cell with a collinear design of the excitation laser beam and gas flow. We provide a model describing the signal dependence on the main operational parameters such as modulation frequency and flow velocity. The results reveal the existence of optimal working regimes, depending on the ratio between modulation frequency and flow velocity. Besides the known signal decrease with higher modulation frequencies [2], we show that for the case with flow, an as-yet undescribed pattern is superimposed. Using water vapor in synthetic air as the target substance, we give a detailed analytical and experimentally supported approach to PTS parameter optimization to increase signal strength and, hence, sensor performance.

## 2. Methods

### 2.1. Fabry–Pérot Interferometer

We used a fiber-coupled, air-spaced Fabry–Pérot etalon for RI-change sensing to measure the concentration of an analyte of interest, which was, in our case, exemplary of H${}_{2}$O. The FPI measuring principle relies on the change of the probe laser beam intensity reflected from the etalon cavity mounted in the sensor head [16]. By using an FPI with a probe beam wavelength λ, an etalon of a fixed length *L* and a certain finesse *F*, the ratio between the reflected and the incoming intensity $I_{R}$/$I_{0}$ depends in good approximation on the refractive index *n* of the medium inside the cavity. This relation is described by [16]:

(1) $$\begin{array}{c} \frac{I_{R}}{I_{0}}=\frac{F\;sin^{2}\left(\frac{2\pi nL}{\lambda }\right)}{1+F\;sin^{2}\left(\frac{2\pi nL}{\lambda }\right)}. \end{array}$$

A modulated laser beam is most commonly used to excite the analyte, creating a thermal wave that emanates from the irradiated and absorbing volume. The change in temperature ΔT alters the fluid RI, as stated by the Clausius–Mossotti Equation [6]:

(2) $$\begin{array}{c} \Delta n=-\left(n_{0}-1\right)\frac{\Delta T}{T_{abs}}, \end{array}$$

with $n_{0}$ and $T_{abs}$ being the RI and the absolute temperature of the unheated fluid, respectively. In our setup, synthetic air with a predefined water vapor concentration was guided through a narrow duct, in which the Fabry–Pérot etalon was integrated. Inside this channel, the excitation laser beam propagated collinearly along with the gas flow and through the etalon.

Increasing the overlap between the probe laser beam and the excitation laser beam would provide advantages resulting from an overall longer optical path length. However, as this study made use of a highly sensitive, commercially available FPI (see Section 2.4) to measure the RI change, the collinear alignment of gas flow and probe beam was impossible due to geometrical constraints.

### 2.2. Analytical Model of the Thermal Evolution

A suitable mathematical description is essential to predict the temperature distribution inside the duct and, as a result, to calculate the signal magnitude at a particular position of interest. To that, the heat-diffusion equation provides a general starting point for describing the thermal evolution of the given application geometry. It describes the relationship between the spatial and temporal temperature distribution T(r,t) for a given, time-dependent heat source Q(r,t) [17]:

(3) $$\begin{array}{c} \frac{\partial T(r,t)}{\partial t}=D\;\nabla^{2}T\left(r,t\right)-v\;\nabla T\left(r,t\right)+\frac{1}{\rho c_{p}}Q\left(r,t\right), \end{array}$$

with *D*, v, ρ and $c_{p}$ being the fluid’s thermal diffusivity, directional velocity, density and heat capacity, respectively.

In Equation (3), the time- and space-dependent temperature change δT can be interpreted as a spatiotemporal convolution, denoted by ⊛, of the delivered thermal power density $Q_{H}$ and the temperature impulse response $\delta T_{point}$ [1]:

(4) $$\begin{array}{c} \delta T\left(r,t\right)=Q_{H}\left(r,t\right)⊛\delta T_{point}\left(r,t\right). \end{array}$$

Solutions for the spatial and temporal temperature response for photothermal excitations can be found in the literature for various configurations [6,17,18]. For the configuration used in this work, we can solve Equation (3) by applying the Green’s function method [17,18]. Given an alignment of the Gaussian excitation laser beam that is collinear with the direction of the moving fluid, the solution is:

(5) $$\begin{array}{cc} T\left(x,y,z,t\right){|}_{y=0}= & \;\frac{\alpha }{\pi \rho c_{p}}\;\int_{0}^{t}P\left(\tau \right)\;R^{\prime }\left(\tau \right)\;\frac{e^{-2z^{2}/\left(a^{2}+8D\left(t-\tau \right)\right)}}{a^{2}+8\;D\;\left(t-\tau \right)}\;\\ \; & \;\cdot \;\left(-erf\left\{\frac{-x+v_{x}\left(t-\tau \right)}{2\sqrt{D(t-\tau )}}\right\}\right.\\ \; & \left.+\;erf\left\{\frac{-x+v_{x}\left(t-\tau \right)+x_{length}}{2\sqrt{D(t-\tau )}}\right\}\right)\;d\tau ,\\ \; & \;\mathrm{for}\;0\leq x\leq x_{length}, \end{array}$$

with α, *a*, $x_{length}$ and *D* being the absorption coefficient, the excitation laser beam 1/$e^{2}$-radius, the total length of the excitation channel and the thermal diffusivity, respectively. The variable $v_{x}$ denotes the gas velocity in the x-direction, and erf is the Gauss error function.

### 2.3. Simulation of the Temperature Response

A highly resolved temperature distribution in the excitation channel is a prerequisite for an analytical description that correctly predicts the experimental data. Our model parameters used in the calculation matched those of the experimental runs (see Section 3.3) as closely as possible.

In the simulation, $v_{x}$ in Equation (5) was described by the velocity profile of a no-slip, fully developed, incompressible and laminar flow inside a rectangular channel. The function P(τ) was the time-dependent optical power irradiated by the excitation laser, which we regarded as sinusoidal. Accordingly, we specified the optical power in Equation (5) as:

(6) $$P\left(t\right)=P_{av}\;\left(1+sin\left(\omega t\right)\right),$$

with $P_{av}$ being the average laser optical power and ω the angular excitation modulation frequency.

The time necessary for the conversion of the absorbed energy to thermal energy is finite. Therefore, the function $R^{\prime }\left(\tau \right)$ in Equation (5) describes the total thermal relaxation impulse response of the excited molecules and considers the delayed rise in temperature upon irradiation. However, as the analyte of interest in this work, H${}_{2}$O, exhibits a fast relaxation rate at high concentrations and in air [19], $R^{\prime }\left(t\right)$ can be approximated by a Dirac delta function $\delta_{0}^{\prime }\left(t\right)$ for the excitation frequencies and water vapor concentration used in this study. A listing of the exact parameter values used in the simulation is provided in the Appendix A. The derivation of Equation (5) is available in the Supplementary File.

**Figure 1:**
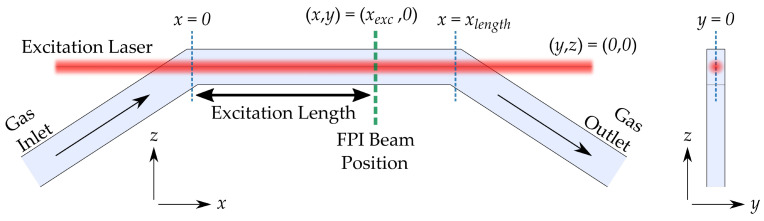
Schematic sketch of the channel geometry with the coordinates of the relevant elements in *x*–*z*-plane (**left**) and *y*–*z*-plane (**right**). The excitation laser beam (red) propagates centrally through the channel and collinear to the gas flow in the area of exposure, i.e., the excitation channel. Both gas duct (blue area) and etalon (positioned at the dashed green line) are rectangular with a size of 1 × 2 mm (width × height). The excitation length (black arrow) spans approximately 10 mm, starting at the transition from the curvature to the horizontal part of the channel and ending at the position of the FPI probe laser beam.

Referring to the specific geometrical arrangement (see Figure 1), the excitation channel’s length, width and height coincides with the respective *x*, *y* and *z* orientation in our analytical description, i.e., the model. Both gas flow and propagation of the excitation laser beam are orientated in the *x*-direction. The FPI probe laser beam is aligned in the *z*-direction at ($x_{exc}$, 0) and exhibits a length that matches the full channel height $x_{height}$ of 2 mm. The excitation length equals $x_{exc}$ and spans approximately 10 mm. Given a sufficient spacing between the position of the FPI at $x_{exc}$ and the downstream end point of the excitation channel $x_{length}$, the second Gauss error function in Equation (5) approaches unity. In such a case, the diffusion of gas, once passed beyond the excitation channel and back to the FPI, is expected to have a negligible impact if the gas velocity is sufficiently high in relation to the thermal diffusivity.

The temperature distribution given by Equation (5) constitutes the core of our theoretical model. It enables the prediction of experimental signal readings from the amplitude of the periodic temperature change. The temperature change stems from the cyclic heating caused by the modulated excitation laser beam and the subsequent cooling caused by the incoming not yet irradiated air. The temperature amplitude ΔT was determined by calculating the average temperature along a line at the position of the probe laser beam, being at (x,y) = ($x_{exc}$, 0) in our layout. To this end, we computed the spatial and temporal temperature distribution for numerous *z*-positions and averaged it over the entire channel height. The heat exchange between the duct walls and the gas was considered negligible. For substantially narrower gas channel diameters (i.e., ≪1 mm), the heat conduction by the channel walls required consideration, as heat accumulated prior to the measuring point could be significantly dissipated along the path length. Further, we omitted a possible attenuation of the laser beam due to its absorption during the passage through the PT cell. However, we assumed only a marginal and, for our investigation, negligible decreasing laser beam power within the excitation channel at this scale. Despite this simplification, the model provided valuable insights into the signal dynamics of a PTS sensor when varying the gas flow velocity and the modulation frequency, respectively (see Section 3.1), and by performing a modulation frequency sweep for various flow rates. These simulated frequency sweeps were compared with experimental ones (see Section 3.3).

### 2.4. Experimental Setup

We used a customized instrument for the RI change measurement (Eta250 Ultra, XARION Laser Acoustics, Vienna, Austria) featuring a fiber-coupled, air-spaced etalon with a size of 1 × 2 mm (width × height) and a removed protective foil. The probe laser beam yielded an output power of 1 mW at 1550 nm and a Gaussian beam profile with a full width at half maximum of 205 μm [8]. The etalon was of a rigid structure and comprised two semitransparent mirrors 2 mm apart from each other. As for the operating point, the instrument automatically adjusted to the near-optimal sensitivity by wavelength-tuning. Referring to Equation (1), we concluded that the reflected intensity, and thus the output signal of the FPI, depended upon the RI of the medium we measured.

Achieving a high target sensitivity and little cross-sensitivity demands a properly selected excitation source. The laser wavelength was required to match an absorption line of the analyte. The excitation laser was generated by a distributed feedback laser diode (NLK1E5GAAA, NEL, Yokohama, Japan) controlled by a laser and TEC driver (ITC4001, Thorlabs, Newton, NJ, USA), which provided a temporally square-wave-modulated optical output power. The current was modulated between its predefined maximum value and a value slightly above the threshold. The laser beam had an average optical power of 13 mW, which was monitored with a thermal power meter (PM16-401, Thorlabs) after the beam passed through the measurement cell. The beam waist was collimated to a 1/$e^{2}$ radius of approximately 0.43 mm. The collimator (F230APC-1310, Thorlabs) and the power meter were placed as closely as possible to the cell windows to reduce the attenuation of the laser beam by the ambient water vapor. In order to determine the appropriate excitation wavelength, we accessed the HITRAN database [20] using the HITRAN Application Programming Interface [21]. We selected the wavenumber to coincide with a rotational–vibrational transition at 7327.7 cm${}^{-1}$, providing a high absorption cross section. In the experimental frequency sweep, we increased the excitation modulation frequency in steps between 50 and 200 Hz. Since the temperature of the laser diode, and thus the wavelength of the beam it emitted, depended on the modulation frequency, the temperature setting on the laser driver was adjusted for each modulation frequency to maintain a constant absorption cross section.

### 2.5. Photothermal Cell

**Figure 2:**
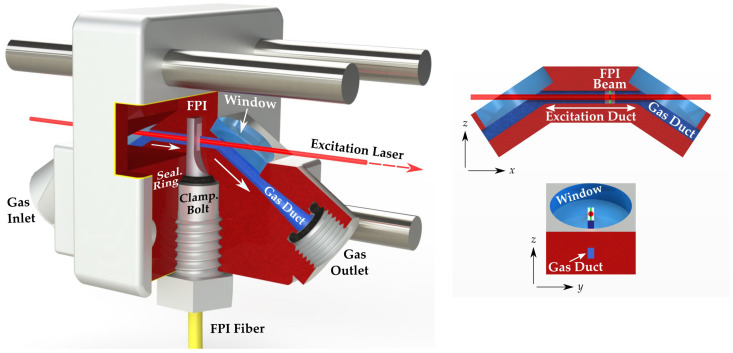
Photothermal cell used for the PTS measurements. The excitation laser beam with a 1/$e^{2}$ radius of 0.43 mm is aligned collinearly to the movement direction of the gas in the excitation channel. The beam enters the cell through a window arranged at the Brewster angle. The fiber-coupled, air-spaced etalon for monitoring the RI is fitted firmly into the channel system and held gas-tight by a clamping bolt and a sealing ring. Areas colored in red and blue symbolize a sectional view through the cell and its gas duct system, respectively, which serves solely for visualization. The FPI probe beam (green) in the upper right sketch is drawn to scale as FWHM for visibility.

A PT cell was designed to guide the entire gas flow through the etalon while allowing for a laser beam irradiation collinear to the flow upstream of the etalon (see Figure 2). Owing to the nature of PTS, matching the modulation frequency to the acoustic resonance of the cell was not required, which provided flexibility in designing and optimizing the PT cell architecture towards individual performance criteria, such as system downsizing or a response time reduction. However, as thermal waves decay considerably faster than acoustic waves [22], irradiation should be performed near the measurement area. The cell channel matched the square aperture of the etalon, providing a seamless duct-to-etalon transition to minimize the generation of flow noise. The inlet and outlet channel sections, along with two N-BK7 windows, were aligned at the Brewster angle (56.4${}^{\circ }$) relative to the excitation laser beam axis. This angle ensured a maximum light transmission into the excitation region (i.e., the horizontally orientated section of the PT cell channel). At the gas-tight openings of the PT cell, the channel was initially kept circular with a 2 mm radius for a seamless transition to the polytetrafluoroethylene (PTFE) tubing. Moving towards the excitation channel, it gradually tapered and changed its geometry to a rectangular profile. The measuring head (etalon) was clamped into the PT cell through a screw-in hollow cylinder that pressed an O-ring seal against the body of the sensor holder, thus fixing it gas-tight. An exact laser beam alignment was required to avoid the irradiation of the channel walls. We chose this channel length as a compromise between a satisfactorily extended excitation length and a laser beam that could still be aligned with sufficient precision.

A vacuum pump combined with critical orifices was used to maintain a constant gas flow through the PT cell. The highest experimentally used mass flow rate was 1.2 standard liters per minute (slm). During the experimental frequency sweep (i.e., Section 3.3), we added a custom-built, Herschel–Quincke (HQ) damper upstream of the cell to attenuate external acoustic noise. For determining the flow noise (see Section 3.2), a mass flow controller (MFC) was used instead of the orifices to adjust the flow through the cell. In this case, the HQ filter was added between the PT cell and the MFC to suppress acoustic interference coming from the vacuum pump.

Acoustic flow noise can, when coinciding with the lock-in frequency, severely impact the PTS performance by raising its signal variance. It may significantly degrade the sensor signal. We tried to keep noise to a minimum by retaining a laminar flow regime and preventing turbulence as much as possible. Although engineering-related influences such as the transitions between individual parts of the gas line (e.g., tubing to cell ports) were contributory factors, the noise power was primarily driven by the gas flow velocity.

### 2.6. Humidity Generation, Signal Processing and Data Analysis

We used H${}_{2}$O as the analyte because of its uncomplicated and safe nature in handling. The enrichment of the prior dry synthetic air with water vapor was accomplished by a custom-built humidity generator, described in the studies by Lang et al. [19]. A custom-built gas dilution unit positioned downstream of the humidity generator was used to adjust the water content of the air further. This gas diluter featured a binary mixing scheme consisting of a series of temperature-stabilized critical orifices of different sizes [23]. We used an H${}_{2}$O volume concentration of 9700 ppm for the frequency sweep in Section 3.3.

**Figure 3:**
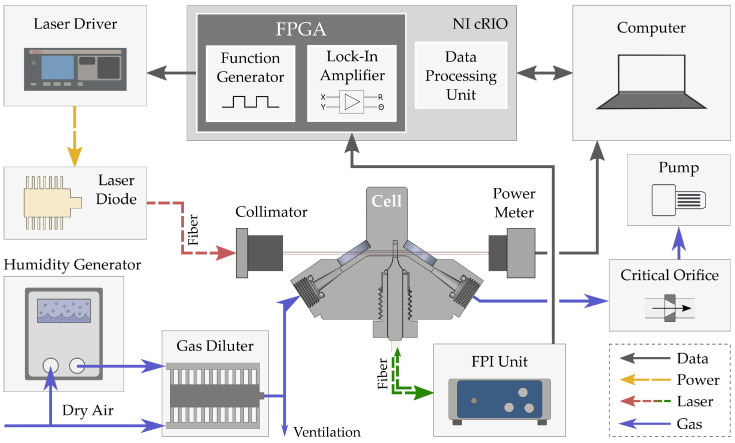
Schematic sketch of the experimental setup with its key components for the modulation frequency sweep. Gas with a predefined water concentration and flow rate is pumped through the cell (solid blue arrows). Periodic temperature change inside the excitation channel, induced by the absorption of the modulated excitation laser (dashed yellow and red arrows), is monitored interferometrically (dashed green arrows). Processing the FPI signal and controlling the laser modulation are accomplished via an FPGA that is part of a real-time computing device (NI cRIO, National Instruments). Solid gray arrows denote the essential data flow.

For data acquisition, the analog FPI output signal was sampled at 51.2 kHz with a 24-bit ADC (NI 9234, National Instruments, Austin, TX, USA). We set the FPI unit to a cut-off frequency of 10 Hz and an output gain of +20 dB. A real-time controller (NI cRIO-9031, National Instruments) performed the data processing, which also served as a function generator for the modulation of the excitation laser beam. In addition, the controller provided the lock-in amplification for detecting the signal’s amplitude and phase at the excitation frequency. We used an integration time of 1 s. The controller and ADC instruments operated on a field programmable gate array (FPGA), and the interaction between the user and the system was accomplished by LabVIEW software (National Instruments). A schematic sketch of the experimental setup is presented in Figure 3.

A quantitative comparison between the experimentally obtained data and the simulation results requires acquiring the correct conversion factor between the signal amplitude *S* (experiment), Δn and ΔT (model). For this purpose, we initially measured the acoustic sensitivity of the FPI using a calibration device (Microtech Type 4000, Microtech Gefell GmbH, Gefell, Germany), obtaining 19.2 mV${}_{ampl}$/Pa${}_{RMS}$ at 1 kHz. We then derived the dependence of the signal amplitude on the RI change by assuming an adiabatic relation between gas pressure and temperature. We employed the Ciddor equations [24] and the conversion factor between RI and gas density ρ [1,25]. Considering the specific output settings of the FPI unit used during our measurements (i.e., a gain of +20 dB), we obtained a sensitivity of 40.6 × 10${}^{9}$ mV${}_{ampl}/n_{ampl}$ at 1 kHz. The further conversion between Δn and ΔT was performed according to Equation (2). The experimental analysis and the analytical signal calculation were executed within a Python environment.

## 3. Results and Discussion

### 3.1. Analytical Model Results

**Figure 4:**
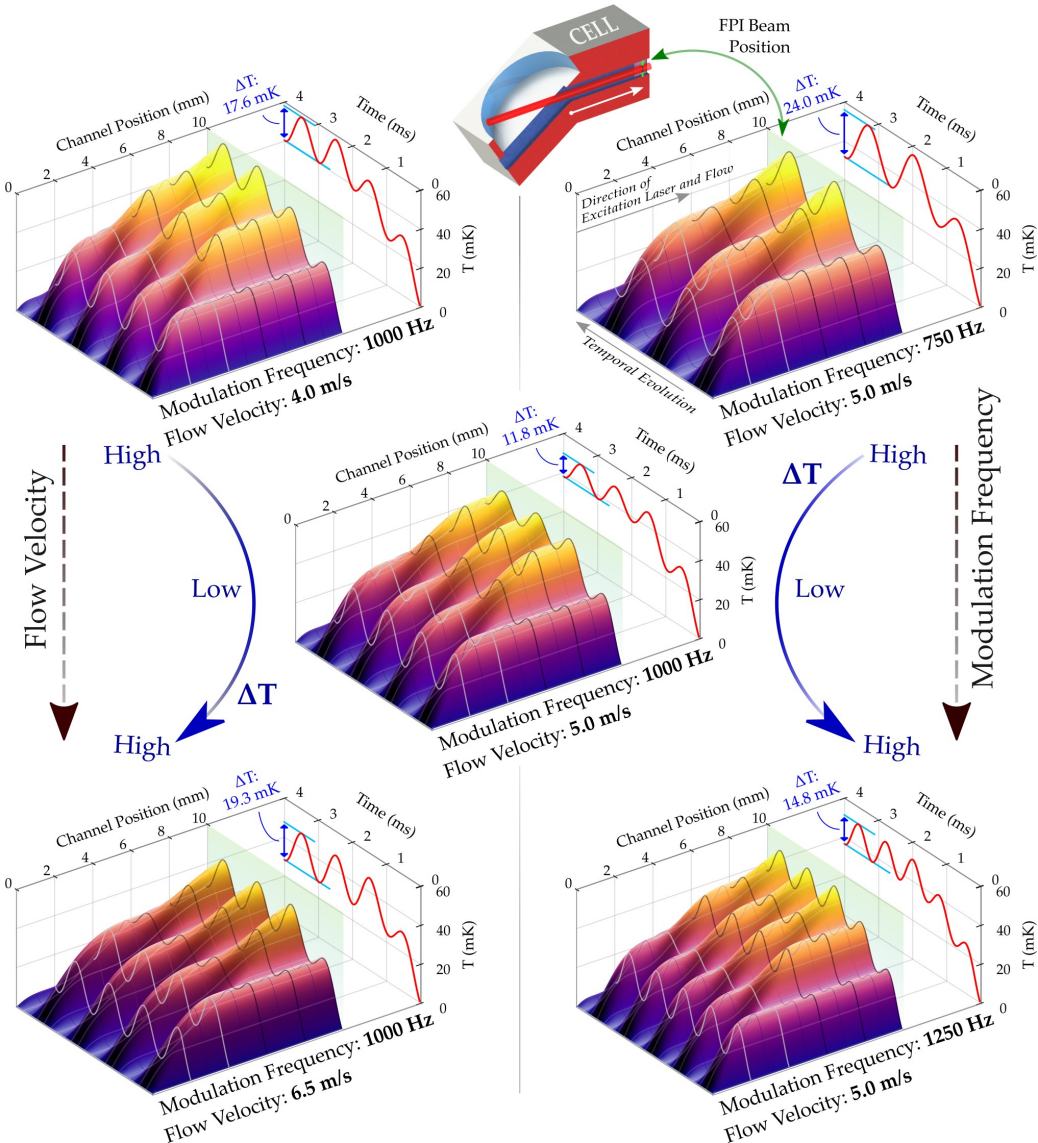
Temporal and positional temperature evolution at the channel centerline for different flow velocities (**left**) and modulation frequencies (**right**). The channel position axis represents the *x*-position inside the excitation channel. The variation in temperature ΔT (plotted at the red line as peak-to-peak) at the position of the FPI beam (translucent green plane) initially decreases towards both a higher flow velocity and modulation frequencies (top to center plot) before increasing again (center to lower plots). The center plot configuration shows an unfavorable relationship between flow velocity and modulation frequency at 1000 Hz and 5.0 m/s by yielding a low ΔT. The illustration on top shows a cross-sectional view of the excitation area of the used PT cell.

Generally, one may expect a monotonically decreasing temperature amplitude towards higher modulation frequencies and flow velocities [2,26]. Our model showed a behavior that deviated from this expectation, as presented in Figure 4. It yields the temporal evolution of the temperature *T* at the centerline of the excitation channel for five different configurations. The 3D plots map the absorption-induced temperature changes for three flow velocities at a constant modulation frequency (left-hand side), and three modulation frequencies at a constant flow velocity (right-hand side), respectively. At the position of the FPI probe laser beam, i.e., after an excitation path of approximately 10 mm, the associated temperature is depicted as a red line in each plot. The sinusoidal temperature amplitude becomes discernible after a certain thermalization period (note that ΔT in Figure 4 represents the peak-to-peak temperature). Our model indicates that the system thermalizes sufficiently at the centerline within tens of milliseconds, even at the lowest modulation frequency and flow rate experimentally used (i.e., 50 Hz at 0.05 slm).

We observe that the temperature amplitude does not decrease monotonically with increasing modulation frequencies or flow velocities. The center plot in Figure 4 (i.e., 1000 Hz and 5.0 m/s), for example, exhibits the lowest ΔT, although it represents the median in terms of both modulation frequency and flow velocity. It is found that ΔT not only depends on the actual modulation frequency and flow rate but, considering the respective excitation length, also on their ratio. An ideal ratio develops on the one hand when the incoming gas is irradiated as entirely as possible over the excitation length during the heating period. Due to the advective cooling after each excitation period, a maximized peak-to-peak temperature difference can be generated for a chosen flow rate by optimizing the excitation frequency. The larger peak-to-peak amplitude then directly relates to a larger RI signal that can be measured this way, yielding a higher signal amplitude.

**Figure 5:**
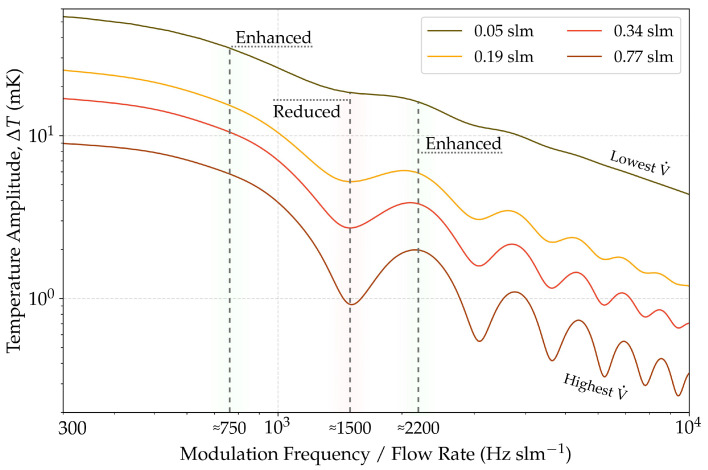
Temperature amplitude as a function of the ratio between modulation frequency and mass flow rate for four selected mass flow rates $\dot{V}$. Areas with greater temperature amplitude and areas with reduced amplitude appear, which are more pronounced towards larger $\dot{V}$. The temperature amplitude is averaged over the entire channel height.

Figure 5 shows the temperature amplitude, averaged over the channel height, plotted as a function of the ratio between modulation frequency and mass flow rate $\dot{V}$ for four different $\dot{V}$. Following the previous findings, cyclic variations in ΔT appear, which are more pronounced towards higher $\dot{V}$. The temperature amplitude decreases towards higher ratios for all flow rates. The respective signal-enhanced and signal-reduced areas have their positions at nearly similar ratios for all flow rates. For low ratios, the distance between the local extreme values of the variations is nearly equidistant (i.e., approximately 750, 1500 and 2200). The positions we considered extreme values are described in Section 3.3. Lower flow rates yielded higher temperature amplitudes at equal ratios. The applied flow rates, i.e., 0.05 to 0.77 slm, corresponded to those used in the experimental run (see Section 3.3).

Figure 5 shows the existence of distinct regions depending on the ratio between modulation frequency and flow rate. The signal-enhanced region with the smallest ratio appears to be the most suitable operational set-point. Thus, keeping the flow rate and modulation frequency as low as possible would be reasonable. However, this ratio require low modulation frequencies that are susceptible to ambient noise. However, care should be taken, since the given ratio values refer to our setup. In particular, the excitation length influences the position and intensity of these regions.

The signal amplitude depends on the input parameters. The parameters that appear particularly interesting are found in the integral part of Equation (5) which is provided in a more general form in Supplementary Materials. Among those are the excitation length $x_{exc}$, the excitation channel’s cross-sectional area, the flow velocity $v_{x}$ and the excitation laser’s radius *a*. Increasing $x_{exc}$ results in higher signal amplitudes and larger distances between the signal peaks and vice versa. A larger cross-sectional area, i.e., the height of the excitation channel *z* times the width of the excitation channel *y*, decreases the flow velocity, if the flow rate is kept constant. A reduced flow velocity increases the signal amplitude and narrows their peaks (see Section 3.3). A circular shape of the excitation channel might enable a higher heating efficiency. However, a circular-shaped excitation channel was not considered due to the rectangular geometry of the used FPI etalon. The signal amplitude further depends on the diameter of the excitation laser beam. Assuming equal irradiance, reducing the laser beam´s diameter yields a higher signal amplitude for all modulation frequencies. This becomes evident when considering an excitation laser beam that is collimated along the channel centerline. In this case, the gas fraction off-center is less irradiated. Since the gas fraction off-center has a lower flow velocity than the gas on the centerline, there is a variation in the ratio between excitation frequency and flow velocity over the channel’s cross-sectional area. A higher dispersion of the ratio across the irradiated cross-sectional area thus reduces the temperature amplitude when it is averaged over the channel height. As a result, narrowing the excitation laser’s diameter reduces the variation in the velocity of the gas fraction that corresponds to the average temperature amplitude, which leads to a higher signal amplitude.

Changes in the gas composition may affect the thermal diffusivity *D* and the relaxation. A higher thermal diffusivity lowers the signal amplitude, since it leads to a better thermal exchange between the gas particles that are irradiated during heating and those that remain unheated during cooling. In addition, a thermal transfer in the radial direction also leads to a reduced signal amplitude due to different flow velocities across the channel. A higher relaxation time decreases the measurable signal amplitude for our used PTS configuration due to the delayed heat release (Supplementary Materials).

### 3.2. Flow Noise

**Figure 6:**
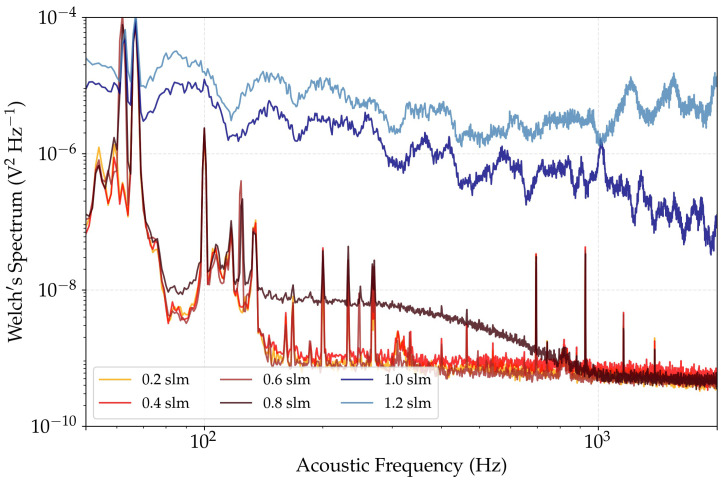
Spectral density estimation (Welch’s spectra) with a blocked excitation laser beam for evaluating the highest reasonable mass flow rate $\dot{V}$ through the cell. Flow rates of 0.6 slm and below show similar noise spectra, implying a laminar flow without significant turbulence. Welch’s spectra for $\dot{V}$ of 0.8 slm and above exhibit a swift noise increase towards higher flow rates. Further experimental runs are thus to be limited to remain below 0.8 slm.

Conducting a spectral density estimation for a broad range of flow rates allows to narrow down the region in which the sensor is expected to remain operable in a low-noise regime. We performed the spectral density estimations using Welch’s method (Welch’s spectrum) from 50 to 2000 Hz for various mass flow rates $\dot{V}$ up to 1.2 slm. Figure 6 shows the resulting Welch’s spectra for an excitation laser beam blocked prior to the PT cell and a Hanning window with a size of 1 s.

For flow rates up to and including 0.6 slm, a 1/f-noise trend with a corner frequency around 150 Hz is identifiable. For these $\dot{V}$, isolated peaks occur across their spectra, possibly due to disturbances caused by the gas diluter or other external factors. The increased noise density at flow rates above 0.8 slm is attributed to a transitioning of the flow from the laminar to the turbulent regime. The two highest rates, i.e., 1.0 and 1.2 slm, exhibit considerably high noise levels over the entire frequency range of interest. These gas flow values thus represent the reasonable upper limits of our analysis for the used configuration. Consequently, we limited $\dot{V}$ to remain below 0.8 slm for this cell geometry.

### 3.3. Excitation Modulation Frequency Sweeps

**Figure 7:**
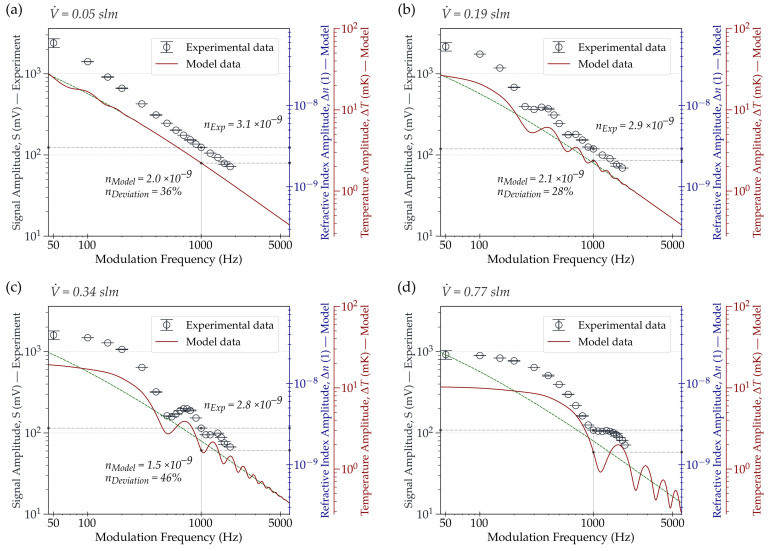
Experimental (circles) and numerical simulation (solid red line) frequency sweep for four selected flow rates. The model features amplitude variations oscillating around a zero-flow baseline (dashed green line) that increase in intensity for higher $\dot{V}$ (from (**a**–**d**)). Variations in amplitude are experimentally not observable for the lowest $\dot{V}$ (**a**) but become distinctly noticeable towards higher $\dot{V}$. Deviations between experiment and model in amplitude (for all $\dot{V}$) and oscillation position (for high $\dot{V}$) are attributable to parameter uncertainties in the model (e.g., flow velocity distribution) and engineering constraints in the experimental setup (e.g., PT cell imperfections). The measured values (in mV) are convertible into Δn and ΔT at 1 kHz via the given sensitivity of the FPI device.

We performed a numerically simulated and an experimental sweep of the excitation laser frequency for different gas flow rates $\dot{V}$. Figure 7 shows the amplitude with respect to the modulation frequency of the model’s temperature (right-hand axis) and the experiment’s signal (left-hand axis). As for the model, it displays the temperature amplitude ΔT in the frequency range from 45 to 6000 Hz for four selected flow rates between 0.05 and 0.77 slm. A zero-flow calculation was added for comparison. The temperature amplitude was calculated by averaging the temperature over the full probe beam length, as described in Section 2.2. Sinusoidal excitation was assumed for both the model and the experiment.

On the experimental side, Figure 7 presents the modulation frequency sweep from 50 towards 2000 Hz in steps of 50 to 200 Hz. The same four flow rates were used in the model. The step size was narrowed at frequencies where we detected a change in the signal amplitude associated with the pattern predicted by the simulation. The model and experiment could be quantitatively compared at 1 kHz using the interferometer calibration. All experimentally acquired data points were background-corrected according to the procedure described by Lang et al. [19].

The simulation results (solid lines) in plots (a) to (d) in Figure 7 correspond to the behavior described in Section 3.1, as they exhibit cyclic variations in ΔT (further referred to as oscillations). The oscillations are centered around a reference baseline trajectory with decreasing oscillation amplitude towards higher frequencies. The baseline, i.e., the zero-flow case, displays the simulated behavior of ΔT over the modulation frequency for a stationary fluid (depicted as a dashed line). It separates into two opposing areas in which the ratio between flow rate and modulation frequency has either a constructive or a destructive effect. Consequently, the sensor operates either in a signal-enhanced or a signal-reduced regime. Both optimum and most subideal settings can be considered as those where the difference of ΔT to the baseline is the largest.

Although the oscillations in the model are found to occur for all non-zero flow rates, they differ in intensity and position. At the lowest $\dot{V}$ of 0.05 slm (a), a weak manifestation of the periodic cycle appears, discernible in the range below 200 Hz. By increasing $\dot{V}$ (b–d), the oscillations intensify with augmenting distances between their peaks. One further notices an amplitude saturation at higher flow rates towards lower frequencies. This results from the fact that below a particular modulation frequency, the total heating time within one excitation period ceases to increase by further lowering the modulation frequency. The saturation marks the point at which the excitation period begins to become substantially larger than the time required for the gas to pass through the excitation area. In such a case, an increasing fraction of the gas becomes exposed to radiation over the entire excitation length during the heating period. Similarly, during the cooling period, an increasing fraction of the gas reaches the FPI without being exposed to radiation. At saturation, the gas heating time stops depending on the modulation frequency and becomes dependent solely on the flow velocity and the excitation length. The respective frequency at which the saturation emerges depends on the gas velocity and the total excitation length. As the flow velocity inside the excitation channel is inhomogeneously distributed, the transition into the saturation frequency range is gradual.

The experimentally obtained signal amplitudes (i.e., the circles in Figure 7) reveal similar dependencies on the ratio between the modulation frequency and $\dot{V}$. When comparing the oscillations from the experiment to those of the model, they appear less intense. While not detectable in the experiment for the lowest flow rate (a), they manifest noticeably at higher flow velocities. The absence of measurable oscillations in the lowest flow regime (a) is attributable to their weak expression and the large-stepped sweep of at least 50 Hz. We detect them distinctly for the first time for a flow rate of 0.19 slm in the modulation frequency range below 1000 Hz (b). For 0.34 slm (c), the signal amplitude oscillates significantly across the entire measured frequency range, though still noticeably weaker than in the model. However, we notice a considerable signal difference of about 25.7% when raising the modulation frequency by 150 Hz from 550 to 700 Hz.

Comparing the experimental and the simulated trajectories, the positions of the oscillations gradually diverge towards higher modulation frequencies. For 0.77 slm (d), a considerable positional shift between experiment and simulation in the range above 1000 Hz is recognizable. We suppose that this discrepancy is mainly attributable to the mismatch between the anticipated and the actual experimental gas velocity distribution inside the PT cell channel. While, in theory, we assumed a fully developed flow in the excitation channel, this condition was not fulfilled in the experiment. Considering our cell channel, gas above a particular flow rate cannot achieve a fully developed flow before the excitation area. Additionally, the channel bending prior to the excitation section causes the gas, owing to its momentum, to be densified towards the upper channel wall. Given these considerations, it is more reasonable to assume an axially asymmetric, not yet fully developed flow profile.

As for the conversion between the model’s and experiment’s units, we observe RI differences at 1 kHz for all flow rates in the lower, double-digit percentage range (i.e., 36, 28 and 46% in (a), (b) and (c), respectively). For the highest flow rate (d), the deviation (i.e., 47%) has a reduced relevance due to the positional offset of the oscillation at 1 kHz.

We attributed the experimentally observed discrepancy in the oscillation intensities compared to those of the simulation primarily to engineering factors and/or potential uncertainties in the model parameters (Table A2). Upon conducting several experimental pretests, we recognized a significant dependence in the manifestation of the oscillations on the measurement setup. In more detail, their appearance decreased substantially with even a slight misalignment between the excitation laser beam and cell channel axis. Moreover, our PT cell suffered from 3D-print-related topographical imperfections, further limiting the accuracy in practice. Additionally, since we were constrained to 1 kHz when converting the model’s and experiment’s units, deviations in amplitude at frequencies other than 1 kHz might have been caused by a changing FPI sensitivity. This analysis considered its sensitivity to be constant across the entire frequency spectrum, which might explain the considerably more pronounced amplitude gap observable in the lower frequency range.

Both simulation and experimental results did not show a monotonous 1/f decrease of the amplitude towards higher modulation frequencies for this PTS configuration. Instead, we found a more complex relationship involving the ratio between modulation frequency and flow rate that superimposed the 1/f trend. Although our model used a simplified representation, it included all essential parameters for providing a rough but reliable estimate of the range in which a favorable configuration for a given modulation frequency or flow rate could be expected. Similar to the simulation, the experimental results revealed that the signal amplitude depended on the modulation frequency to flow rate ratio. Although the measured oscillations were less pronounced compared to the simulation, it was experimentally shown that a changing modulation frequency could affect the sensor signal to a greater extent than the 1/f trend suggested. Simultaneously, keeping the flow rate constant is essential for ensuring and maintaining the accuracy of a PTS sensor during operation. Our data strongly advise that a densely meshed parameter sweep should be performed in any case.

## 4. Conclusions

Our work revealed a dependency of the PTS signal amplitude on the ratio between the excitation laser’s modulation frequency and the gas flow velocity. The findings were based on simulations and experiments describing oscillations in the signal originating from the gas flowing through the excitation channel. Our model described this behavior and provided a good starting point for obtaining the optimal modulation frequency and flow rate settings to achieve the highest signal intensity. The experimentally observed oscillations were less pronounced compared to those of the model. We attributed these deviations to simplifications in the assumed flow profile, uncertainties in model parameters and the experimental setup used.

The observation that the signal intensity is highly related to the ratio between flow velocity and excitation frequency has considerable implications for PTS configuration with a collinear gas flow to excitation laser arrangement. One is the requirement for a sufficiently small step size in the frequency sweep to find the flow-enhanced areas and obtain the optimal signal intensity. A further important aspect comes from the substantial change in signal intensity caused by comparatively small frequency and/or flow rate changes. These changes may significantly affect the reading of the sensor during use. For practical sensor applications, this requires that the gas flow in particular—along with the excitation laser frequency—be kept constant. Any change in flow rate, for example caused by flow regulators, might cause erroneous data.

We conclude that for an FP-based PTS sensor with a collinear arrangement between the excitation laser beam and the gas flow direction, the signal intensity is distinctly sensitive to changes in flow velocity or excitation frequency. Choosing the parameters without proper care might place the sensor in a flow-reduced working regime, whereas proper parameter tuning enables the sensor to operate in the flow-enhanced one. We provided a simple model that highlighted the existence of signal-enhanced domains and estimated the settings required to access these. Our experimental results confirmed the model predictions.

## Data Availability

The data sets used in this study are available from the corresponding author on reasonable request.

## Supplementary Materials

The following is available online at https://www.mdpi.com/article/10.3390/s22197148/s1, Supplementary File: Derivation of Equation (5).

## Abbreviations

The following abbreviations are used in this manuscript:

PT	photothermal
PTS	photothermal spectroscopy
RI	refractive index
MFC	mass flow controller

## Appendix A. Model Parameters

A complete register of the input parameters used to calculate the model temperature from Equation (5) is given in the tables below. Table A1 holds the primary parametric values with unit and description. Table A2 contains secondary parameters derived from the primary ones. We obtained the coefficients $c_{p}$, $v_{ha}$ and κ in Table A2 using the open-source Python library CoolProp [27], optimized to determine thermophysical properties.

**Table A1:** Listing of the primary parameters for the calculation of the theoretical temperature amplitude ΔT.

Symbol	Value	Unit	Description
$x_{height}$	2	mm	Excitation channel height
$x_{width}$	1	mm	Excitation channel width
$x_{length}$	16	mm	Excitation channel length
$x_{exc}$	10.5	mm	Excitation length
*a*	0.43	mm	Excitation laser 1/e${}^{2}$ radius
$P_{av}$	13 × 10^−3^	W	Average optical exc. laser power
Hz	50 to 2000	Hz	Exc. laser modulation frequency
$\dot{V}$	0.05 to 0.77	slm	Gas mass flow rate
$X_{H_{2}O}$	9.7 × 10^3^	1	H${}_{2}$O mole fraction
$\sigma_{H_{2}O}$	5.75 × 10^−24^ [21]	m^2^ molec^−1^	H${}_{2}$O absorption cross section at 7327.7 cm${}^{-1}$
$p_{cell}$	101,325	Pa	Absolute gas pressure
$T_{cell}$	298.25	K	Absolute gas temperature
$k_{B}$	1.381 × 10^−23^	J K^−1^	Boltzmann’s constant

**Table A2:** Listing of the secondary parameters for the calculation of the theoretical temperature amplitude ΔT, derived from Table A1.

Symbol	From	Approx. Value	Unit	Description
$c_{p}$	*f*($T_{cell}$, $p_{cell}$, $X_{H_{2}O}$) [27]	1.012 × 10^3^	J kg^−1^ K^−1^	Mixture specific heat per unit dry air
$v_{ha}$	*f*($T_{cell}$, $p_{cell}$, $X_{H_{2}O}$) [27]	0.876	m^3^ kg^−1^	Mixture volume per unit humid air
κ	*f*($T_{cell}$, $p_{cell}$, $X_{H_{2}O}$) [27]	2.697 × 10^−2^	W m^−1^ K^−1^	Mixture thermal conductivity
$\rho_{0}$	$v_{ha}^{-1}$	1.142	kg m^−3^	Density of humid air
$N_{0}$	$p_{cell}$ $T_{cell}^{-1}$ $k_{B}^{-1}$	2.461 × 10^25^	molec m^−3^	Total number concentration
*D*	κ $\rho_{0}^{-1}$ $c_{p}^{-1}$	2.334 × 10^−5^	m^2^ s^−1^	Thermal diffusivity
ω	2πHz	314 to 12,566	rad s^−1^	Exc. laser angular frequency
α	$N_{0}$ $X_{H_{2}O}$ $\sigma_{H_{2}O}$	1.373	m^−1^	Absorption coefficient at 7327.7 cm${}^{-1}$
