# Supplementary material for: Flow-Enhanced Photothermal Spectroscopy
Source: Sensors (Basel). 2022 Sep 21;22(19):7148. doi: 10.3390/s22197148 (PMC9570771; doi:10.3390/s22197148)
Supplement: Supplementary file 1 [file sensors-22-07148-s001.zip › supplementary.pdf]

Supplementary Materials: Derivation of Equation (5)

The model's time-resolved, three-dimensional temperature distribution $T(x,y,z,t)$ used in the main text is deduced in the following. The approach is analogous to that for two dimensions, described concisely in [2] and in more details in chapter three in [1]. The variables whose meaning is not described below are explained in the main text.

Here, the heating source, i.e., a laser beam, propagates along with the moving fluid in the x-direction. Thermal diffusivity is considered in all directions. The derivation starts with the temperature differential equation, which is found in the main text and writes as:

$$\frac{\partial T(x,y,z,t)}{\partial t} = D\,\nabla^2 T(x,y,z,t) - v_x\,\nabla T(x,y,z,t) + \frac{1}{\rho c_p} Q(x,y,z,t). \tag{1}$$

The Green's function method solves Equation (1), yielding:

$$T(x,y,z,t) = \int_0^\infty \int_{-\infty}^\infty \int_{-\infty}^\infty \int_{-\infty}^\infty Q(\xi,\eta,\theta,\tau)\; G(x/\xi; y/\eta; z/\theta; t/\tau)\; \mathrm{d}\xi \mathrm{d}\eta \mathrm{d}\theta \mathrm{d}\tau, \tag{2}$$

with G being the Green's function and Q the heat. The latter is defined as:

$$Q(x,y,z,t) = \frac{2\alpha E_0}{\pi a^2\, t_0} e^{-2(y^2+z^2)/a^2} \quad \text{for } 0 \le t \le t_0 \wedge x_0 \le x \le x_1, \tag{3}$$

with E_0 and t_0 being the total energy and time duration of one laser pulse, respectively. For $t > t_0$, $Q(x,y,z,t)$ is zero.

The differential equation for the Green's function is:

$$-\,D\,\nabla^2_{xyz}\,G + v_x \frac{\partial G}{\partial x} + \frac{\partial G}{\partial t} = \frac{1}{\rho\, c_p}\;\delta(x-\xi)\;\delta(y-\eta)\;\delta(z-\theta)\;\delta(t-\tau), \tag{4}$$

with the boundary conditions being:

$$\begin{aligned} G(\pm\infty/\xi;\ y/\eta;\ z/\theta;\ t/\tau) &= 0,\\ G(x/\xi;\ \pm\infty/\eta;\ z/\theta;\ t/\tau) &= 0,\\ G(x/\xi;\ y/\eta;\ \pm\infty/\theta;\ t/\tau) &= 0,\\ G(x/\xi;\ y/\eta;\ z/\theta;\ 0/\tau) &= 0. \end{aligned} \tag{5}$$

Fourier transform of Equation (4) is:

$$(\omega_x^2+\omega_y^2+\omega_z^2)D\;G_F - i\omega_x v_x G_F + \frac{\partial G_F}{\partial t} = \frac{1}{(2\pi)^{3/2}\rho\, c_p}\; e^{i(\omega_x\xi+\omega_y\eta+\omega_z\theta)}\;\delta(t-\tau). \tag{6}$$

Laplace transform of Equation (6) gives:

$$(\omega_x^2+\omega_y^2+\omega_z^2)D\;G_{FL} - i\omega_x v_x G_{FL} + s\,G_{FL} = \frac{1}{(2\pi)^{3/2}\rho\, c_p}\; e^{i(\omega_x\xi+\omega_y\eta+\omega_z\theta)}\;e^{-s\tau}. \tag{7}$$

The solution of Equation (7) is:

$$G_{FL} = \frac{e^{i\omega_x\xi}\;e^{i\omega_y\eta}\;e^{i\omega_z\theta}\;e^{-s\tau}}{(2\pi)^{3/2}\rho\, c_p[D(\omega_x^2+\omega_y^2+\omega_z^2) - i\omega_x v_x + s]}. \tag{8}$$

The inverse Laplace transform of G_{FL} gives:

$$G_F = \frac{e^{i\omega_x \xi}\, e^{i\omega_y \eta}\, e^{i\omega_z \theta}\, H_\tau(t)}{(2\pi)^{3/2} \rho\, c_p} e^{\left(i\omega_x v_x - (\omega_x^2 + \omega_y^2 + \omega_z^2) D\right)(t-\tau)}, \tag{9}$$

with $H_\tau(t)$ being the Heaviside function:

$$H_\tau(t) = \begin{cases} 0 & \text{for } 0 \le t < \tau \\ 1 & \text{for } t \ge \tau. \end{cases} \tag{10}$$

The inverse Fourier transform of G_F yields the Green's function:

$$\begin{aligned} G = \frac{H_\tau(t)}{(2\pi)^{3/2} \rho\, c_p} \frac{1}{(2\pi)^{3/2}} & \int_{-\infty}^{\infty} e^{i\omega_x(\xi + v_x(t-\tau))} e^{-\omega_x^2 D(t-\tau)} e^{-i\omega_x x}\, \mathrm{d}\omega_x \\ & \cdot \int_{-\infty}^{\infty} e^{i\omega_y \eta} e^{-\omega_y^2 D(t-\tau)} e^{-i\omega_y y}\, \mathrm{d}\omega_y \\ & \cdot \int_{-\infty}^{\infty} e^{i\omega_z \theta} e^{-\omega_z^2 D(t-\tau)} e^{-i\omega_z z}\, \mathrm{d}\omega_z. \end{aligned} \tag{11}$$

For solving the integrals in Equation (11), the convolution theorem is used, which, in the following exemplarily for x, has the form:

$$\begin{aligned} \int_{-\infty}^{\infty} F(\omega_x) G(\omega_x) e^{-i\omega_x x}\, \mathrm{d}\omega_x &= \int_{-\infty}^{\infty} \mathscr{F}_{\omega_x}^{-1}\{F(\omega_x)\}(\lambda)\ \mathscr{F}_{\omega_x}^{-1}\{G(\omega_x)\}(x-\lambda)\, \mathrm{d}\lambda \\ &= \int_{-\infty}^{\infty} f(\lambda)\, g(x-\lambda)\, \mathrm{d}\lambda. \end{aligned} \tag{12}$$

Applying the convolution theorem, the first integral in Equation (11) writes as:

$$\begin{aligned} & \int_{-\infty}^{\infty} \mathscr{F}_{\omega_x}^{-1}\left\{e^{-\omega_x^2 D(t-\tau)}\right\}(\lambda)\ \mathscr{F}_{\omega_x}^{-1}\left\{e^{i\omega_x(\xi + v_x(t-\tau))}\right\}(x-\lambda)\, \mathrm{d}\lambda \\ &= \int_{-\infty}^{\infty} \frac{1}{\sqrt{2}\sqrt{D(t-\tau)}} e^{-\lambda^2/(4D(t-\tau))} \sqrt{2\pi}\, \delta((x-\lambda) - v(t-\tau) - \xi)\, \mathrm{d}\lambda \\ &= \frac{\sqrt{\pi}}{\sqrt{D(t-\tau)}} e^{-(x-\xi-v(t-\tau))^2/(4D(t-\tau))}. \end{aligned} \tag{13}$$

The second integral in Equation (11) writes as:

$$\begin{aligned} & \int_{-\infty}^{\infty} \mathscr{F}_{\omega_y}^{-1}\left\{e^{-\omega_y^2 D(t-\tau)}\right\}(\lambda)\ \mathscr{F}_{\omega_y}^{-1}\left\{e^{i\omega_y \eta}\right\}(y-\lambda)\, \mathrm{d}\lambda \\ &= \int_{-\infty}^{\infty} \frac{1}{\sqrt{2}\sqrt{D(t-\tau)}} e^{-\lambda^2/(4D(t-\tau))} \sqrt{2\pi}\, \delta((y-\lambda) - \eta)\, \mathrm{d}\lambda \\ &= \frac{\sqrt{\pi}}{\sqrt{D(t-\tau)}} e^{-(y-\eta)^2/(4D(t-\tau))}. \end{aligned} \tag{14}$$

The third integral solves equally to the latter for ($y \to z, \eta \to \theta$).

The Green's function in a simpler form is obtained by substituting the solutions of the Equations (13) to (14) back into Equation (11):

$$
\begin{aligned}
G = \; & \frac{H_\tau(t)}{(2\pi)^3 \rho\, c_p} \, \frac{\pi^{3/2}}{(D(t-\tau))^{3/2}} \, e^{-(x-\xi-v(t-\tau))^2/(4D(t-\tau))} \\
& \qquad\qquad\qquad\qquad e^{-(y-\eta)^2/(4D(t-\tau))} \, e^{-(z-\theta)^2/(4D(t-\tau))} \\
= \; & \frac{H_\tau(t)}{8\pi^{3/2} \rho\, c_p (D(t-\tau))^{3/2}} \, e^{-((x-\xi-v(t-\tau))^2 - (y-\eta)^2 - (z-\theta)^2)/(4D(t-\tau))}.
\end{aligned}
\tag{15}
$$

Substitution of G from Equation (15) and Q from Equation (3) into Equation (2) yields the result:

$$
\begin{aligned}
T(x,y,z,t) = & \int_0^{t_0} \frac{2\alpha E_0}{\pi a^2\, t_0} \, \frac{1}{8\pi^{3/2} \rho\, c_p (D(t-\tau))^{3/2}} \\
& \cdot \int_{-\infty}^{\infty} \int_{-\infty}^{\infty} \int_{x_0}^{x_1} e^{-((x-\xi-v(t-\tau))^2 - (y-\eta)^2 - (z-\theta)^2)/(4D(t-\tau))} \, e^{-2(\eta^2+\theta^2)/a^2} \; \mathrm{d}\xi \mathrm{d}\eta \mathrm{d}\theta \mathrm{d}\tau \\
= & \int_0^{t_0} \frac{\alpha E_0}{4\pi^{5/2} \rho\, c_p\, a^2\, t_0 (D(t-\tau))^{3/2}} \int_{-\infty}^{\infty} \int_{-\infty}^{\infty} \int_{x_0}^{x_1} e^{-(x-\xi-v(t-\tau))^2/(4D(t-\tau))} \\
& \cdot \; e^{-2\eta^2/a^2} \, e^{-(y-\eta)^2/(4D(t-\tau))} \, e^{-2\theta^2/a^2} \, e^{-(z-\theta)^2/(4D(t-\tau))} \; \mathrm{d}\xi \mathrm{d}\eta \mathrm{d}\theta \mathrm{d}\tau \\
= & \int_0^{t_0} \frac{\alpha E_0}{4\pi^{5/2} \rho\, c_p\, a^2\, t_0 (D(t-\tau))^{3/2}} \sqrt{\pi} \, \sqrt{D(t-\tau)} \\
& \cdot \left(-erf\left\{ \frac{-x+v(t-\tau)+x_0}{2\sqrt{D(t-\tau)}} \right\} + erf\left\{ \frac{-x+v(t-\tau)+x_1}{2\sqrt{D(t-\tau)}} \right\} \right) \\
& \cdot \int_{-\infty}^{\infty} \int_{-\infty}^{\infty} e^{-2\eta^2/a^2} \, e^{-(y-\eta)^2/(4D(t-\tau))} \, e^{-2\theta^2/a^2} \, e^{-(z-\theta)^2/(4D(t-\tau))} \; \mathrm{d}\eta \mathrm{d}\theta \mathrm{d}\tau \\
= & \int_0^{t_0} \frac{\alpha E_0}{4\pi^2 \rho\, c_p\, a^2\, t_0 D(t-\tau)} \, 4\pi a^2 D(t-\tau) \, \frac{1}{a^2 + 8D(t-\tau)} \\
& \cdot \left(-erf\left\{ \frac{-x+v(t-\tau)+x_0}{2\sqrt{D(t-\tau)}} \right\} + erf\left\{ \frac{-x+v(t-\tau)+x_1}{2\sqrt{D(t-\tau)}} \right\} \right) \\
& \cdot \; e^{-2(y^2+z^2)/(a^2+8D(t-\tau))} \; \mathrm{d}\tau \\
= & \int_0^{t_0} \frac{\alpha E_0}{\pi \rho\, c_p\, t_0\, (a^2 + 8D(t-\tau))} \cdot e^{-2(y^2+z^2)/(a^2+8D(t-\tau))} \\
& \cdot \left(-erf\left\{ \frac{-x+v(t-\tau)+x_0}{2\sqrt{D(t-\tau)}} \right\} + erf\left\{ \frac{-x+v(t-\tau)+x_1}{2\sqrt{D(t-\tau)}} \right\} \right) \; \mathrm{d}\tau \\
& \text{for } t \geq t_0 \wedge x_0 \leq x \leq x_1,
\end{aligned}
\tag{16}
$$

with erf being the Gauss error function.

We are considering a time-dependent input power source, e.g., a modulated laser beam. Thus the time-independent term E_0/t_0, i.e., P_0, is substituted by the time-dependent term $P(t)$. We define $\tilde{T}(x,y,z,t) = T(x,y,z,t) \,/\, P_0$, and express $T(x,y,z,t)$ as convolution of $P(t)$ and $\tilde{T}(x,y,z,t)$:

$$
T(x,y,z,t) = (P \circledast \tilde{T}(x,y,z))(t) = \int_0^t P(\tau) \, \tilde{T}(x,y,z,t-\tau) \; \mathrm{d}\tau. \tag{17}
$$

This leads to the following expression:

$$T(x,y,z,t) = \frac{\alpha}{\pi\rho\, c_p} \int_0^t P(\tau)\, \frac{e^{-2(y^2+z^2)/(a^2+8D(t-\tau))}}{a^2+8D(t-\tau)} \cdot \left(-erf\left\{ \frac{-x+v(t-\tau)+x_0}{2\sqrt{D(t-\tau)}} \right\} + erf\left\{ \frac{-x+v(t-\tau)+x_1}{2\sqrt{D(t-\tau)}} \right\} \right) \mathrm{d}\tau \quad \text{for } x_0 \le x \le x_1. \tag{18}$$

We further include $R'(t)$ into (18), which is a function of the target- and fluid-specific excitation relaxation time τ_E and accounts for the delayed heat release from the excited molecules:

$$R'(t) = \frac{e^{-t/\tau_E}}{\tau_E}. \tag{19}$$

Combining this with the definition of x_0 as zero leads to the final expression for the temperature distribution found in the main text:

$$T(x,y,z,t) = \frac{\alpha}{\pi\rho\, c_p} \int_0^t P(\tau)\, R'(\tau)\, \frac{e^{-2(y^2+z^2)/(a^2+8D(t-\tau))}}{a^2+8D(t-\tau)} \cdot \left(-erf\left\{ \frac{-x+v(t-\tau)}{2\sqrt{D(t-\tau)}} \right\} + erf\left\{ \frac{-x+v(t-\tau)+x_1}{2\sqrt{D(t-\tau)}} \right\} \right) \mathrm{d}\tau \quad \text{for } 0 \le x \le x_1. \tag{20}$$

References

1. Sell, J. *Photothermal Investigations of Solids and Fluids*; Elsevier: Amsterdam, The Netherlands, 2012.
2. Rose, A.; Vyas, R.; Gupta, R. Pulsed photothermal deflection spectroscopy in a flowing medium: A quantitative investigation. *Appl. Opt.* **1986**, *25*, 4626–4643.
